# Rapid change in the ciprofloxacin resistance pattern among *Neisseria gonorrhoeae* strains in Nuuk, Greenland: time to reconsider preventive and treatment strategies

**DOI:** 10.3402/ijch.v74.26916

**Published:** 2015-05-05

**Authors:** Anne Skjerbæk Rolskov, Karen Bjorn-Mortensen, Gert Mulvad, Peter Poulsen, Jørgen Skov Jensen, Michael Lynge Pedersen

**Affiliations:** 1 Department of Gynaecology and Obstetrics, Hillerød Hospital, Hillerød, Denmark; 2 Department of Epidemiology Research, Statens Serum Institut, Copenhagen, Denmark; 3 Greenland Center for Health Research, Institute of Nursing and Health, University of Greenland, Nuuk, Greenland; 4 Queen Ingrid Health Care Center, Nuuk, Greenland; 5 The Central Laboratory, Queen Ingrid Hospital, Nuuk, Greenland; 6 Microbiology and Infection Control, Statens Serum Institut, Copenhagen, Denmark

**Keywords:** *Neisseria gonorrhoeae*, sexually transmitted infections, antimicrobial resistance, ciprofloxacin resistance

## Abstract

**Objectives:**

Sexually transmitted infections (STIs), including infections with *Neisseria gonorrhoeae* (GC), are highly incident in Greenland. Since January 2011, GC testing has been performed on urine with nucleic acid amplification tests (NAATs) by strand displacement amplification (Becton Dickinson ProbeTec). Monitoring of GC antibiotic susceptibility by culture was introduced in Nuuk in 2012. Until 2014, no cases of ciprofloxacin-resistant GC strains were reported. In this paper, we report the finding of ciprofloxacin-resistant GC and describe the most recent incidence of GC infections in Greenland.

**Methods:**

The number of urine NAATs and culture-positive swabs from January to October 2014 were obtained from the Central Laboratory at Queens Ingrid's Hospital in Nuuk and stratified on gender, place and period of testing. Incidence rates were estimated as number of urine NAAT * (12/10) per 100,000 inhabitants. Men in Nuuk with a positive NAAT for GC were encouraged to provide a urethral swab for culture and susceptibility testing.

**Results:**

From January to October 2014, a total of 5,436 urine GC NAATs were performed on patients from Nuuk and 9,031 from the rest of Greenland. Of these, 422 (8%) and 820 (9%) were positive, respectively. From January to August, 6 (15%) cultures from Nuuk were ciprofloxacin resistant while in September and October, 26 (59%) were ciprofloxacin resistant (p<0.01). In total, 35 (40%) of 88 culture-positive isolates showed ciprofloxacin resistance. GC incidence in Nuuk was 3,017 per 100,000 inhabitants per year, compared to 2,491 per 100,000 inhabitants per year in the rest of Greenland.

**Conclusion:**

Within a short period, a rapid and dramatic change in ciprofloxacin susceptibility among GC strains isolated in Nuuk was documented and recommendation for first line treatments has changed. Continued monitoring and rethinking of primary and secondary preventive initiatives is highly recommended in this high GC incidence setting.

Sexually transmitted infections (STIs), including infections with *Neisseria gonorrhoeae* (GC), are incident in Greenland (1). Different diagnostic, treatment, and preventive strategies have been used over time. Years ago, penicillin-resistant GC strains emerged, leading to a change in the treatment regimen to a single dose of ciprofloxacin. Since 2011, diagnosis of GC in Greenland has been performed on urine with nucleic acid amplification tests (NAATs) by strand displacement amplification (Becton Dickinson ProbeTec) without testing for antibiotic susceptibility. However, a pilot study performed in Nuuk in 2012, reported full ciprofloxacin susceptibility among 35 GC isolates (1). An additional 100 GC isolates collected in 2012 and 2013 were fully susceptible (Unpublished data). Since then, monitoring of ciprofloxacin susceptibility has continued among men in Nuuk. Testing has not been performed routinely outside Nuuk. No cases of ciprofloxacin-resistant GC isolates were reported until the beginning of 2014, when a few resistant cases appeared. This paper describes a sudden increase in ciprofloxacin-resistant GC cases in September 2014 and the most recent incidence of GC infections in Greenland.

## Methods

The number of urine NAATs and culture-positive swabs from January to October 2014 were obtained from the Central Laboratory at Queens Ingrid's Hospital in Nuuk and stratified on gender, place of testing and whether testing was done before or after 1 September 2014. Background population as per 1 July 2014 (2) was used for estimation of incidence rates as number of urine NAAT * (12/10) per 100,000 inhabitants per year.

Initially, only men in Nuuk with a positive urine NAAT for GC or suspected GC treatment failure were further encouraged to provide a urethral swab for culture and ciprofloxacin susceptibility testing, with methods described in details elsewhere (1). From October 2014, susceptibility testing was intensified to include cervical swabs from women with positive NAATs. Chi-square test was used to compare frequencies, with a P-value<0.05 as level of significance.

## Results

A total of 5,436 urine NAATs were performed in Nuuk within the first 10 months of 2014. Of these, 422 (8%) tests were GC positive, corresponding to an incidence of 3,017 per 100,000 inhabitants per year. In comparison, among 9,031 urine NAATs from the rest of Greenland, 820 (9%) were positive corresponding to an incidence of 2,491 per 100,000 inhabitants per year. Eighty-five GC isolates were obtained from patients from Nuuk. Of these, 32 (38%) showed ciprofloxacin resistance. The first ciprofloxacin-resistant GC isolate in Greenland after introducing ciprofloxacin susceptibility testing in 2012 was found in January 2014 and 5 more were observed up until August. Three ciprofloxacin-resistant isolates were found in September and 23 (64%) such isolates were found in October. As seen in Table I, this was a significant change from 15% (n=6) during the first 8 months to 59% (n=26) during the last 2 months (p<0.01). In addition, 3 GC isolates were cultured from the rest of Greenland, all ciprofloxacin resistant. Among 190 positive urine NAATs from men in Nuuk, GC cultures were positive in 64 cases (34%).

**Table I:** Number of urinary nucleic acid amplification tests (NAATs) and cultured swabs for GC in Greenland within 10 months (January–October) of 2014 stratified on gender, place of testing and period

	NAATs				Cultured swabs			
		
	All		Men		All		Men	
				
	Tests, N	Positive, % (n)	Tests, n	Positive, % (n)	Positive, N	Resistant, % (n)	Positive, n	Resistant, % (n)
Nuuk^a^								
Total	5,436	8 (422)	1,748	11 (190)	85	38 (32)	64	31 (20)
January–August	4,201	7 (291)	1,333	10 (129)	41	15 (6)	38	13 (5)
September–October	1,235	11 (131)	415	15 (61)	44	59 (26)	26	58 (15)
Rest of Greenland^b^								
Total	9,031	9 (820)	3,346	12 (396)	3	100 (3)	3	100 (3)
January–August	7,890	7 (584)	2,556	11 (282)	1	100 (1)	1	100 (1)
September–October	1,141	21 (236)	790	14 (114)	2	100 (2)	2	100 (2)

a Population, n=16,786 (men, n=8,812);

b Population, n=39,509 (men, n=20,930).

## Discussion

This paper demonstrates a rapid and dramatic change in ciprofloxacin susceptibility among GC isolates in Nuuk within a short period. This may reflect transmission in sexual networks through unprotected sex. The percentage of ciprofloxacin-resistant GC isolates is now in the same range as reported from other European countries (1) Thirty-four percent of positive NAATs had a corresponding positive culture, indicating that only a minority of men participated in the resistance monitoring program. Previous data suggested that 52–75% positive NAATs yielded a positive culture (1). However, although limited, the monitoring for GC resistance captured the shift in ciprofloxacin susceptibility. This has led to a change in GC treatment to a single dose of 500 mg ceftriaxone i.m. in combination with 2 g azithromycin orally in accordance with current European guidelines (3). However, use of broad-spectrum antibiotics in GC treatment has a risk of developing antibiotic resistance among other pathogenic bacteria, and monitoring of antibiotic susceptibility is recommended to catch newly introduced resistance. Actions towards a future STI strategy including rethinking of primary and secondary preventive initiatives, diagnostic procedures and treatment are highly recommended in this high GC incidence setting.

## References

[1] Pedersen ML, Clausen-Dichow P, Poulsen P, Nyborg H, Jensen JS (2013). Low prevalence of ciprofloxacin-resistant *Neisseria gonorrhoeae* in Nuuk, Greenland. Sex Transm Dis.

[2] Statistics Greenland Population, 2014 Population 1st July.

[3] Bignell C, Unemo M (2013). 2012 European guideline on the diagnosis and treatment of gonorrhoea in adults. Int J STD AIDS.

